# Nutrition assessment and geriatric associated conditions among free living elderly people in Birjand, East of Iran: a cross-sectional study

**DOI:** 10.1186/s12877-021-02518-x

**Published:** 2021-10-30

**Authors:** Fatemeh Hoseinzadeh-Chahkandak, Mehran Rahimlou, Fatemeh Salmani, Elham Ansarifar, Mitra Moodi, Farshad Sharifi, Tayebeh Zeinali

**Affiliations:** 1 Social Determinants of Health Research Center, Department of Public health, School of Health, Birjand University of Medical sciences, Birjand, Iran; 2 Department of Nutrition, School of Medicine, Zanjan University of Medical sciences, Zanjan, Iran; 3 Social Determinants of Health Research Center, Department of Epidemiology and Biostatistics, School of Health, Birjand University of Medical Sciences, Birjand, Iran; 4 Cardiovascular Diseases Research Center, Birjand University of Medical Sciences, Birjand, Iran; 5 Elderly Health Research Center, Endocrinology and Metabolism Population Sciences Institute, Tehran University of Medical Sciences, Tehran, Iran

**Keywords:** Nutrition assessment, Aged, Malnutrition, Nutritional status, Association

## Abstract

**Background:**

Few data are available on the nutritional status of Iranian geriatric population. The present study aimed to determine the nutritional status of older adults’ population in Birjand, East of Iran.

**Methods:**

The community-based cohort study was performed on older adults population (60 years and older) living in urban and rural areas of Birjand. The mini nutritional assessment (MNA) questionnaire was used to assess nutritional status. Anthropometric and biochemical evaluation were also performed for all of the participants.

**Results:**

A total of 1417 geriatric person were enrolled in this study, which, most of them were female (51.9 %). According to MNA tool, most of the participants (73.3 %) had normal nutrition (MNA score ≥ 24). Malnourished (MNA score < 17) and at high risk of malnutrition (MNA score: 17- 23.5) were constituted 0.9 % and 25.8 % of the participants, respectively. Marital status, occupation, period of education and family member were associated with nutritional status. Multiple logistic regression showed that with increase of body mass index (BMI) (OR = 0.96), education years (OR = 0.95), hemoglobin (OR = 0.86) and lymphocyte (OR = 0.98), the odds of malnutrition decreased, but with increase of age the odds (1.03) of malnutrition is also elevated.

**Conclusions:**

MNA could successfully forecast the risk of malnutrition and malnourished people. Sociodemographic factors are associated with the nutritional status.

## Background

Due to the development of health services and rising life expectancy, the mean age of the population in different countries has ﻿increased [1, 2]. In some countries, such as Japan, a large part of the country’s population is older adults [3]. According to the national organizations reports, by 2025 in developing countries, the population of the older adults will reach more than 840 million [4]. In the latest national assessment of the Iranian population, 14.7 % of the Iranian population is older adults and according to statistical forecasts, by 2050, more than 23 % of the Iranian population (equivalent to 26 million people) will expected to be older adults [5, 6].

One of the most important issues of the older adults, especially in poor and low-income countries, is malnutrition [7]. The results of studies have shown that depending on the health status of the older adults and their mobility, the prevalence of malnutrition is between 15 % and 85 % [6]. Malnutrition observed in the older adults is generally protein energy malnutrition and can cause other side effects such as anemia, weakened muscle activity, reduced bone density, impaired immune function, poor wound healing and depression [8].

Aging causes significant changes in various organs of the body, especially the gastrointestinal tract. These changes include decreased salivation, difficulty swallowing, gastrointestinal upset, and constipation that affect food intake. On the other hand, some social factors such as loneliness, dementia and depression also reduce food intake [9].

Various tools have been designed and evaluated for malnutrition screening in adults over the years. Mini nutritional assessment (MNA) is one of detailed and valid methods developed for assessing and evaluating the nutritional condition of older adults that is used in the many of studies [10–13]. The tool assesses a variety of items, including anthropometric, nutritional, and health status, and gives each person an overall score. Based on the overall score, individuals are classified from a range of ‘well nourished’ (scores higher than 24) to ‘at risk for undernutrition’ (scores from 17 to 23.5) and ‘undernourished’ (scores lower than 17) [14].

Considering the importance of assessing the extent of malnutrition among the older adults and considering that in the southern and eastern regions of Iran for reasons such as low income, drought and social factors, the nutritional conditions of the older adults are unsuitable, the present study designed to assess the severity of malnutrition in the older adults population in Birjand with MNA tool.

## Methods

### Study design and population

The information required for this study was recorded among the participants in the Birjand cohort study, which was a community based prospective cohort study with at least 10 years follow up of participants. Details of the method of this cohort have already been published [1]. In summary, this community-based cohort study was performed on an older adults population (60 years and older) living in urban and rural areas of Birjand, a city in eastern Iran.

**Fig. 1:**
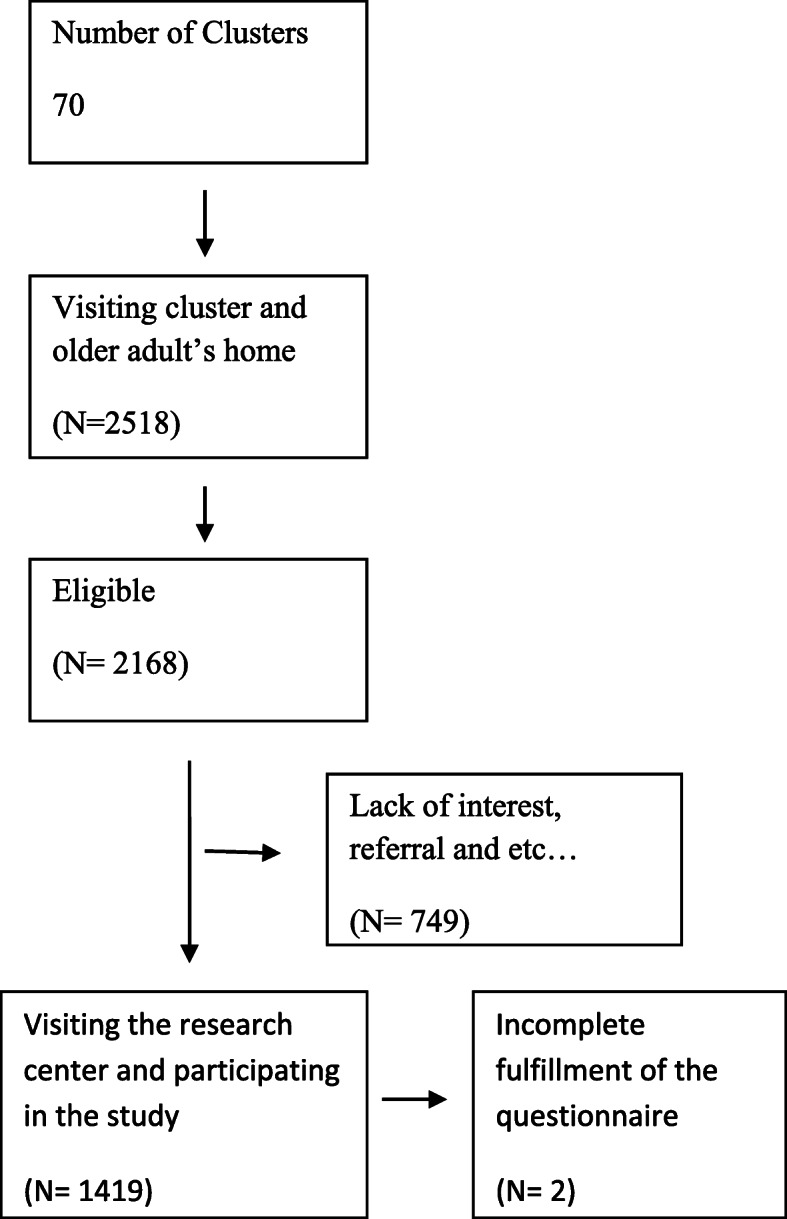
Flow diagram of participants selection

 Multistage stratified cluster sampling was used for participant’s selection. For participant’s selection, using postal codes, seventy clusters were identified and 20 elderlies from each cluster were randomly selected. Older adults living in rural areas were also randomly selected from ten rural health centers. All older adults aged 60 years and above residents of Birjand were included in the study. The study excluded those who had completely bedridden, serious cognitive diseases like Alzheimer’s or Parkinson’s, or who had a very low standard of living (less than 6 months). Figure 1 shows the flow diagram of participant selection.

### Sociodemographic assessment

To obtain sociodemographic information, detailed questionnaires were used (e.g., sex, date of birth, phone numbers, years of academic learning, and job). The required information concerning the diagnosis of previous illness was obtained from self-reported or informant-reported clinicians.

### Nutritional questionnaire

The MNA questionnaire was used to assess nutritional status, which contained 18 questions in four general areas included: 1-Anthropometric measurements (weight, height, and weight loss); 2- Global assessment (six questions related to lifestyle, medication, and mobility); 3- Dietary questionnaire (eight questions, related to number of meals, food and fluid intake, and autonomy of feeding); 4- Subjective assessment (self-perception of health and nutrition).

Finally, based on the scores obtained, people are classified into three subgroups in terms of nutritional status, including participants with good nutritional status scored higher than 24, those at risk of malnutrition (score from 17 to 23.5) and the older adults with malnutrition with a score < 17 [15].

### Anthropometric measurements

Anthropometric measurements included: weight, height and body mass index (BMI). Participants’ weight and height were evaluated with seca measurement device (GmbH, Germany). BMI, often known as Quetelet’s index, was computed as weight (kg) divided by height squared (m^2^). The BMI was also categorised using World Health Organization (WHO) guidelines.

### Laboratory sampling and examination

Blood samples were obtained from all subjects after 12 h of fasting in order to evaluate blood variables. Blood samples were immediately transferred to the central laboratory of Birjand University of Medical Sciences. Clot blood samples were centrifuged in a 6000 SM centrifuge and the serum was extracted. The serum was separated into two micro-tubes, one for log-term bio-banking and the other for further biochemistry tests. Cell blood count (CBC) was analyzed by automated haematology analyzer. Serum concentration of total cholesterol was also measured by Enzymatic (CHOD-PAP) colorimetric method. Serum concentration of fasting blood sugar (FBS) was also evaluated by the enzymatic colorimetric method (Pars Azmoon, Tehran, Iran).

### Statistical analysis

SPSS 20 was used to analyze the data (SPSS for Windows, SPSS Inc., Chicago, IL, USA). All values are reported as mean ± standard deviation (SD) for continuous variables or frequency (%) for categorical variables. The frequency of baseline variables including age-groups, body mass index (BMI) and MNA score were evaluated using Person’s Chi-square or Fisher`s exact test among participants. We also used the One-Way ANOVA to compare MNA score between well-nourished, at risk of malnutrition, and malnourished participants. The simple and multiple logistic regression analysis were used to estimate the relationship between some demographic, blood parameters factors and nutritional status. P-values less than 0.05 were considered to be statistically significant.

## Results

A total of 1417 geriatric persons (age > 60) were participated in present study. The age of participants was 69.73 ± 7.56 (mean ± SD). Most of the participated geriatrics were females (51.9 %). According to MNA evaluation, most of the participants (1039 − 73.3 %) had normal nutrition (MNA score ≥ 24). Malnourished (MNA score < 17) and at high risk of malnutrition (MNA score: 17- 23.5) were constituted 0.9 % (13) and 25.8 % (365) of the participants, respectively. Since, the frequency of malnourished people was low; they were mixed with at risk of malnutrition to evaluate the effect of socio-demographic and other variables on the status of nutrition. The mean of MNA score was 24.56 ± 2.30 with a range of eight to 30. Table 1 presents the demographic status of the geriatrics according to the nutritional status. According to sex, males were less malnourished (malnourished and at high risk of malnutrition) than females (*p* < 0.001). Marital status of normal and malnourished people was also different (*p* < 0.05). Nutritional status and occupation (*p* < 0.001) were significantly associated. Moreover, period of education and family member were lower in malnourished and at risk people (*p* < 0.05).

**Table 1:** Demographic profiles of the geriatrics according to the nutritional status

Variable		Normal (*n* = 1039)	Malnourished and at high risk (*n* = 378)	*P* value
Sex	female	494	241	0.001
	male	545	137	
Marital status	married	868	285	0.009
	single	16	5	
	widow	155	88	
Occupation	retired	428	98	0.001
	employee	5	1	
	farmer	31	9	
	free	107	25	
	housewives	420	222	
	unemployed	46	23	
	other	2	0	
		Mean ± SE		
Education period		4.83 ± 0.16	2.96 ± 0.22	0.0001
Number of children		5.01 ± 0.063	5.23 ± 0.10	0.078
Number of family member		2.62 ± 0.037	2.43 ± 0.06	0.008

The average consumption of meat, chicken, egg and legumes were significantly higher in normal nourished people (*p* < 0.05), but fish usage was the same in normal and malnourished people (*p* > 0.05) (Table 2). Among the dairy, the average consumption of cheese and yogurt were significantly higher in normal nourished people (*p* < 0.0001). Vegetables consumers were higher in normal nourished people (*p* < 0.0001).

**Table 2:** Mean of consumption of different food groups in older adults population (meals/week)

Type of food	Mean ± SD		P value
	Normal (*n *= 1039)	Malnourished and at high risk (*n* = 378)	
Meat	1.81 ± 1.13	1.62 ± 1.14	0.005
Chicken	2.16 ± 1.06	1.94 ± 1.19	0.001
Fish	0.28 ± 0.54	0.25 ± 0.52	0.316
Egg	2.47 ± 1.65	2.15 ± 1.70	0.001
Legumes	1.03 ± 0.64	0.83 ± 0.71	0.000
Milk	2.92 ± 2.53	2.65 ± 2.48	0.07
Cheese	4.84 ± 2.18	3.71 ± 2.32	0.000
Yogurt	3.74 ± 2.24	2.78 ± 2.24	0.000
Fruit	3.58 ± 1.97	3.34 ± 2.53	0.061
Vegetables	3.14 ± 2.32	2.30 ± 2.34	0.000

The blood parameters, including red blood cell count (RBC), hemoglobin [1], hematocrit (HCT), mean corpuscular volume (MCV), mean corpuscular hemoglobin (MCH), mean corpuscular hemoglobin concentration (MCHC), and platelet, had significant difference (*p* < 0.05) according to the nutritional status as all of them except platelet was higher in the normal people (Table 3). Despite the similarity of white blood cell count (WBC) in two groups, neutrophil and lymphocyte were different (*p* < 0.05). The average of cholesterol and FBS was similar in both groups of normal and malnourished people (*p* > 0.05) (Table 3). According to Table 3, the history of previously diagnosed disease, including diabetes mellitus (DM), hypertension (HTN), gout (GOT), and hyperlipidemia (HLP) were significantly associated with status of nutrition, but non-alcoholic fatty liver (NFL) showed no association with nutritional status. Some factors, such as age, sex, education year, family member, RBC, HB, HCT, neutrophil, lymphocyte and BMI were entered into the logistic regression model to determine the best predicting model for malnutrition with forward method.

**Table 3:** Blood parameters (Mean ± SE) and history of diseases (frequency) of older adults

	Mean ± SE		
	Normal (*n* = 1039)	Malnourished and at high risk (*n* = 378)	
WBC (E3/µL)	6.23 ± 0.05	6.39 ± 0.09	0.095
RBC (E6/µL)	4.89 ± 0.01	4.77 ± 0.03	0.0001
Hemoglobin (g/dl)	14.36 ± 0.05	13.75 ± 0.09	0.0001
HCT (%)	42.02 ± 0.12	40.56 ± 0.23	0.0001
MCV (fL)	86.10 ± 0.16	85.30 ± 0.30	0.014
MCH (pg)	29.42 ± 0.07	29.07 ± 0.20	0.039
MCHC (%)	34.16 ± 0.05	33.89 ± 0.08	0.005
Platelet (E3/µL)	225.35 ± 2.05	237.03 ± 4.07	0.005
Neutrophil (%)	52.75 ± 0.30	54.08 ± 0.53	0.025
Lymphocyte (%)	37.72 ± 0.28	36.53 ± 0.50	0.035
Monocyte (%)	6.62 ± 0.08	6.42 ± 0.14	0.236
Eosinophil (%)	2.97 ± 0.05	2.90 ± 0.07	0.507
Cholesterol (mg/dl)	198.12 ± 1.32	194.87 ± 2.10	0.199
FBS (mg/dl)	109.27 ± 1.05	113.14 ± 2.30	0.082
	n(%)		
DM	238(22.9 %)	113(29.9 %)	0.007
HTN	417(40.1 %)	189 (50 %)	0.001
GOT	6 (0.6 %)	9(2.4 %)	0.003
HLP	308(29.7 %)	149(39.6 %)	< 0.001
NFL	107(10.3 %)	50(13.3 %)	0.20

According to Table 4 in simple logistic regression, age (OR = 1.04; CI = 1.023–1.055), sex, BMI, education year, HB and lymphocyte were significantly associated with the odds of malnutrition (*p* < 0.05). Multiple logistic regression with simultaneous evaluation of all predictors determined that with increase of age the odds ratio of malnutrition is also elevated (OR = 1.03). Females are more likely to develop malnutrition (OR = 1.72). With increase of BMI (OR = 0.96), education years (OR = 0.95), HB (OR = 0.86) and lymphocyte (OR = 0.98), the odds of malnutrition was decreased.

**Table 4:** Simple and multiple logistic regression models for malnourished older adults population

	Simple Logistic regression					Multiple Logistic regression				
Predictors	beta	SE	OR	CI95 %		beta	SE	OR	CI95 %	
				0.025	0.975				0.025	0.975
Age (years)	0.038	0.008	1.04	1.023	1.055	0.030	0.01	1.03	1.014	1.048
BMI (kg/m^2^)	-0.034	0.012	0.967	0.944	0.990	-0.036	0.013	0.96	0.940	0.989
Education period	-0.083	0.014	0.921	0.896	0.946	-0.050	0.015	0.95	0.924	0.979
Sex	0.663	0.124	1.941	1.523	2.473	0.54	0.153	1.72	1.271	2.316
HB (%)	-0.240	0.040	0.787	0.728	0.850	-0.154	0.043	0.86	0.788	0.934
Lymphocyte	-0.014	0.007	0.986	0.974	0.999	-0.015	0.007	0.98	0.972	0.998

## Discussion

The older adults defines as people aged ≥ 60 years old by the United Nations, and the category also applies to Iranian population [16]. In order to decrease the risk of malnutrition in the older adults, documented data on their nutritional status requires [17]. The MNA had been validated for the prognosis of the nutrition situation in older adult’s studies in different population from community level to hospitalized patients [11, 18–23].

Most of the participants of the current study had normal nutrition. Abdollahzade et al. (2018) investigated the status of nutrition in older adults referred to Shiraz Jahanidegan Council and observed that most of the participants (86.1 %) were categorized as older adults with normal nutritional status, 12.8 % and 1/1 % of them were at risk of malnutrition, and malnourished, respectively [24], which agreed to the present study. Prevalence of malnutrition in the Iranian older adults reported in various studies [6, 12, 25–28] from 0 % in Tabriz [29] to 12 % in Khorasan-Razavi provinces [30]. According to a meta-analysis in Iranian elderly, the overall prevalence of malnutrition was estimated as 12.2 %. It was 21.6 % and 9.2 % in the older adults residents of nursing homes and those living in home, respectively [6]. Other countries reported similar values of malnutrition as 2 % in Taiwan [31], 3.3 % in Spanish older adults [32], 14 % in South India [33]. About 27.2 % of community-dwelling older male were at risk of malnutrition and malnourished by MNA in Turkey [34]. It was reported as 29.7 % for female in Turkey [35] which was similar to the current study. In a Malaysian study, 55.2 % of the hospitalized older adults were malnourished [36], which was higher than the current study that may be related to performing the current study at the community level not the hospital. According to a case-control study in the older adults diabetic patients, 6.5 % of participants (12 % of diabetic and 1 % of non-diabetic patients) were malnourished, 48.5 % (56.6 % of diabetic and 40.5 % of non-diabetic patients) and 45 % (31.5 % of diabetic and 58.5 % of non-diabetic patients) of them were at risk of malnutrition and normal, respectively [37]. The changes in aging process including decreases taste, smell and physical activity, problems of dental health are among the main promoters of malnutrition [38]. Aging by changing in gastrointestinal system, including decrease of gastric acid secretion, limited absorption of macro and micronutrients could leads to malnutrition [18, 39].

The results of the current study showed that females are more malnourished than men. It was also observed in different studies [12, 13, 27, 30, 34, 35, 37, 40–42]. It showed that women had a higher rate of risk of malnutrition. Marital status had a significant effect on the nutrition of older adults. This finding was supported by other studies that showed better eating habits and appetite in married group than single (single or widowed) ones [27, 30, 43]. There was also a significant association between malnutrition and occupation in the present study. Abdollahzade et al. (2018) didn’t find any significant relationship between age, gender, marital status, former occupation, and malnutrition [24]. Some studies showed that unemployed older adults were more malnourished than employed ones [12, 30, 41, 44]. It may be related to activity of older adults in the community and having social support and also the better status of income of employed older adults.

Furthermore, geriatric people with lower education years were more malnourished than higher periods of education. Kucukerdonmez et al. (2017) investigated the nutritional status of older adults according to their living situation and observed that higher education increased MNA score. Other studies of prevalence of malnutrition in older adults showed that a significant association existed between the educational period of older adults and malnutrition [12, 24, 26, 27, 30, 41, 44]. It may be related to higher knowledge of the educated people regarding healthy lifestyle and searching for good nutrition. The findings of the present study showed that geriatric people who live with the family had normal nutrition. Rate of malnutrition was higher in the older adults participants that lived alone in Turkey [13] and Iran [12, 25, 30]. Loneliness can be lead to less appetite and decrease of food consumption. Socially active people had a higher rate of consuming food [45]. Eating food with someone accompany increased the feel of healthiness [46]. This finding was observed in other studies as well that geriatric people who lived with their family had normal nutrition [47].

Results of the present study showed that the average of consumption of proteins, milk and dairy products and vegetables were higher in older adults with normal nutrition status. A higher rate of fiber’s use significantly reduced the risk of frailty in older adults and protein intakes were not related with the risk of frailty [48]. Moreover, higher intakes of milk drinks reduced the risk of frailty in Chinese older adults, but this association was disappeared for vegetables, fruits, meat and fish products dietary patterns [49]. Elderly’s malnutrition could be avoided by increasing the consumptions of foods enriched in carotenoids and vitamins like vegetable foods [50].

The blood parameters, such as RBC, HB, HCT, MCV, MCH, MCHC, and lymphocyte were lower in malnourished older adults, but platelet and neutrophil were higher. It was proposed that HB and lymphocyte are suitable indicator of malnutrition [51–53]. Age-correlated immune dysregulation like reduced lymphocyte proliferation was related to malnutrition [54]. Other studies revealed a significant correlation between MNA and lymphocyte count [53, 55]. It was observed that none of biochemical marker could propose a suitable screening test for nutrition [56]. Diabetes, hypertension and cardiovascular disease were among the prevalent chronic diseases of malnourished older adults in Bakhtiari et al. [12] while in the current study, diabetes and hypertension were seen frequently in malnourished older adults. Lower food intakes and metabolic disorders of geriatric people had negative effects on the health status of them and lead to progress of diseases [12]. Age and educational period had association with poor nutrition [12] like the current study. Kucukerdonmez et al. (2017) reported age, gender, education period and BMI as the predictors of MNA scores [13] which was according to current study.

## Strength and limitation

The first strength of this study is the community based study on the large sample of representativeness. This helps to clarify the nutrition status of older adults accompanying with their laboratory assessment. We can mention the data of this study as a baseline for programming the interventional nutritional studies as the second strength point of the study. The most important limitation of the study is the absence of frail and bedridden older adults. Moreover, the details of food intakes were not assumed.

## Conclusions

According to present study, older adults in South-Khorasan province had a good nutritional status; however there were some malnourished people that need to provide nutritional support. Females, single, illiterate, unemployed, and older adults who lived alone were more malnourished. Increase of age elevates the odds of malnutrition. Females are more likely to develop malnutrition. Elevation of BMI, education years, HB and lymphocyte decreased the odds of malnutrition. MNA could forecast the risk of malnutrition and malnourished people and suggested to be employed as a monitoring questionnaire for nutrition of the older adults.

## Data Availability

The datasets used and analyzed in this study are available from the corresponding author on reasonable request.

## Abbreviations

MNA: mini nutritional assessment
BMI: body mass index
HB: hemoglobin
WHO: World Health Organization
CBC: Cell blood count
FBS: fasting blood sugar
SD: standard deviation
RBC: red blood cell count
HCT: hematocrit
MCV: mean corpuscular volume
MCH: mean corpuscular hemoglobin
MCHC: mean corpuscular hemoglobin concentration
WBC: white blood cell count
DM: diabetes mellitus
HTN: hypertension
GOT: gout
HLP: hyperlipidemia
NFL: non-alcoholic fatty liver
